# Molecular Characterization of African Swine Fever Viruses from Outbreaks in Peri-Urban Kampala, Uganda

**DOI:** 10.1155/2019/1463245

**Published:** 2019-04-01

**Authors:** Frank Norbert Mwiine, Joseph Nkamwesiga, Christian Ndekezi, Sylvester Ochwo

**Affiliations:** College of Veterinary Medicine, Animal Resources and Biosecurity, Makerere University, P.O. Box 7062, Kampala, Uganda

## Abstract

African swine fever (ASF) is an infectious transboundary disease of domestic pigs and wild swine and is currently the most serious constraint to piggery in Uganda. The causative agent of ASF is a large double-stranded linear DNA virus with a complex structure. There are twenty-four ASFV genotypes described to date; however, in Uganda, only genotypes IX and X have been previously described. Inadequate ASF outbreak investigation has contributed to the delayed establishment of effective interventions to aid the control of ASF. Continuous virus characterization enhances the understanding of ASF epidemiology in terms of viral genome variations, extent, severity, and the potential source of the viruses responsible for outbreaks. We collected samples from pigs that had died of a hemorrhagic disease indicative of ASF. DNA was extracted from all samples and screened with the OIE recommended diagnostic PCR for ASF. Partial B646L (p72), full-length E183L (p54) genes, and CVR region of the P72 gene were amplified, purified, and sequenced. Web-based BLAST and MEGA X software were used for sequence analysis. ASF was confirmed in 10 of the 15 suspected pig samples. Phylogenetic analysis confirmed circulation of genotype IX by both full-length E183 (p54) and partial B646L (p72) gene sequencing. Intragenotypic resolution of the CVR region revealed major deletions in the virus genome, in some isolates of this study. The marked reduction in the number of tetrameric tandem repeats in some isolates of this study could potentially play a role in influencing the virulence of this particular genotype IX in Uganda.

## 1. Background

African swine fever (ASF) is a viral hemorrhagic disease of both domestic pigs and different species of wild swine [1]. This disease is caused by a large double-stranded DNA virus with an icosahedral symmetry. African swine fever virus (ASFV) is the lone member of genus* Asfivirus* and class* Asfarviridae *[2]. There is a broad range in ASFV virulence, and it has been reported that some strains have morbidity and case fatality rates that approach 100%[3]. Since its first description in Kenya [4], the disease has been reported in various countries around the world, recently in China, but remaining endemic in Sardinia, East Africa, and Southern Africa, where it represents a major threat for development of the pig industry [5, 6].

The ASF has periodically been reported in most parts of the world including Western Europe and outside Africa, where ASF was eventually eradicated in most cases by the slaughter of infected and in-contact animals, safe carcass disposal, good sanitation, disinfection, quarantine, and the prevention of contact with wild suids and infected ticks. ASF, however, remains endemic on the island of Sardinia (Italy) in the Mediterranean. ASF is presently endemic in a greater part of sub-Saharan Africa with the island of Madagascar inclusive [7], where it is maintained by both a sylvatic cycle involving ticks associated with wild suids and a domestic cycle involving direct pig to pig transmission in different regions [8].

African swine fever viral genome has around 170 to 195kbp depending on the isolate [9]. This DNA codes for more than 50 different proteins. Using the ASFV major capsid protein p72 that is coded for by the B646L gene, twenty-four ASFV genotypes have been identified and named I to XXIV [10–13].

In Uganda, the previously characterized ASFV viruses have been classified into genotype IX [14, 15] and genotype X [16]. Sequencing of the complete p54 gene has been shown by earlier studies to offer additional, intermediate resolution when typing the ASF viruses [17]. Studies have indicated that the use of the three regions of the ASFV DNA, p72, p54, and CVR, to characterize ASFV is much sufficient despite the presence of many other markers [15].

In this study, we set out to identify the African swine fever virus genotypes isolated from an outbreak in domestic pigs from two farms located in the outskirts (suburbs) of Kampala city so as to contribute sequence data for molecular epidemiology studies and to provide further understanding into ASF disease outbreak patterns by mapping of viral genotypes to geographical regions they circulate within.

## 2. Methods

### 2.1. Study Area

This study was conducted in Muyenga and Kanyanya, both Kampala city suburbs in central Uganda. This was in response to a reported hemorrhagic disease outbreak that killed many domestic pigs with clinical characteristics suggestive of ASF around August 2015.

### 2.2. Study Design and Study Description

This was a cross-sectional study in which outbreak samples from the periurban setting areas of Kanyanya and Muyenga were collected and properly archived. Eight pigs were obtained from Kanyanya while seven pigs were from Muyenga. The fifteen carcasses were transported from the two outbreak sites to Makerere University College of Veterinary Medicine, Animal Resources and Biosecurity (COVAB).

### 2.3. Sample Collection

Samples were collected from pigs that died of a hemorrhagic disease typical of ASF in Muyenga and Kanyanya, Kampala city suburbs-Uganda. During postmortem, approximately four milliliters of whole blood was collected from dead pigs, into labeled tubes containing anticoagulant (EDTA). The collected blood was later on aliquoted into labeled cryovials. Samples including lymph nodes, spleen, tonsils, nasal swabs, and kidney were collected from dead pigs into well-labeled falcon tubes containing 10mls of PBS and stored at -80°C until DNA was extracted.

### 2.4. Extraction of Genomic DNA

DNA was extracted from all the different samples collected from respective pigs. Extraction was done using the DNeasy Blood & Tissue Kit (Qiagen, Germany). 200*µ*l of whole blood and about 400mg of tissue was used as starting material and followed the manufacturer's protocol.

### 2.5. The PCR Amplification of Viral DNA

A 257bp region consistent to the central portion of the* p72* gene was amplified using the Office International des Epizooties (OIE) (Paris, France) recommended ASF diagnostic primers: Primer 1 DiagP1 (5'-ATGGATACCGAGGGAATAGC-3') and Primer 2 DiagP2 (5'-CTTACCGATGAAAATGATAC-3') [19, 20]. Genotyping primers which amplify a 478bp C-terminal region of the p72 gene, p72-U (5'-GGCACAAGTTCGGACATGT-3'), and p72-D (5'-GTACTGTAACGCAGCACAG-3') as described previously ([10, 12] were also used. Additionally, the whole gene encoding the p54 protein was amplified using the primers PPA722 (5'-CGAAGTGCATGTAATAAACGTC-3') and PPA89 (5'-TGTAATTTCATTGCGCCACAAC-3') flanking a 676bp DNA fragment [15]. The B602L (CVR) gene was amplified with the primer pairs CVR-FL1 (5'-TCGGCCTGAAGCTCATTAG-3') and CVR –FL2 (5'-CAGGAAACTAATGATGTTCC-3') as previously described [10, 21]. The DNA amplification of each of the three genotypic genes was performed in a 50*µ*l volume in the presence of 25*µ*l of 2X MyTaq™ Red mix (Bioline, UK), 1.5*µ*l of each 10*µ*M primer concentration, 19.5*µ*l of PCR water, and 2.5*µ*l of DNA extract. The PCR positive control was ASFV isolate Ug12.Kampala3, genotype IX [14].

The PCR amplification program for diagnostic PCR included an initial denaturation at 95°C for 7 minutes, followed by 30 cycles of final denaturation at 94°C for 1 minute, annealing at 55°C for 1 minute, extension at 72°C for a 1 minute, and final extension at 72°C for 10 minutes. The amplification program for the genotyping PCR included an initial denaturation at 96°C for 5 minutes followed by 40 cycles of final denaturation at 95°C for 12s, annealing at 50°C for the 30s, and extension at 72°C for 30s as adapted previous studies [14].

Amplification products were resolved on a 1% agarose gel and run against 100bp *λ* DNA-Hind III and *ϕ*x174 DNA-Hae III mix (Finnzymes, USA) DNA ladder at 125V in 1X Tris-Acetic acid-EDTA (TAE) buffer containing 0.5*µ*g/ml ethidium bromide for 35 minutes. The ethidium bromide stained gels were visualized using the UVIEC™ gel documentation system.

### 2.6. Genotyping of ASFV Using the B646L (p72), E183L (p54), and Central Variable Region

Gene (PCR) amplification products were resolved against a molecular weight marker on a 1% agarose gel to confirm the expected band size. The PCR products were then purified using Qiagen PCR purification kit (Qiagen, USA). The purified products were sequenced by INQABA BIOTEC (South Africa). The raw (ABI) sequences were viewed and edited using BioEdit (Ibis Biosciences, Carlsbad, CA) to remove any ambiguous sequences and the poor reads. Using the NCBI based BLASTn program, similarity search of the obtained nucleotide sequences against other ASFV sequences at GenBank database was performed. The nucleotide sequences of the B646L (p72), E183L (p54), and CVR genes from this study were later deposited in the GenBank under accession numbers KY611350-KY611358, KY688445-KY688454, and MH491288-MH491296, respectively. The evolutionary history was inferred by the Maximum Likelihood method based on the Kimura 2-parameter model [22] using MEGA X software program [23]. The CVR amino acid multiple sequence alignment was done using MUSCLE housed at the EMBL-EBI web server [24].

### 2.7. Data Analysis and Interpretation

Nucleotide sequence data were analyzed following standard procedures and appropriate versions of BioEdit for manual sequence alignment and editing ambiguous nucleotides while MEGA X software package was used to construct the phylogenetic trees.

## 3. Results

### 3.1. Confirmatory Diagnosis of ASFV Using PCR

**Figure 1:**
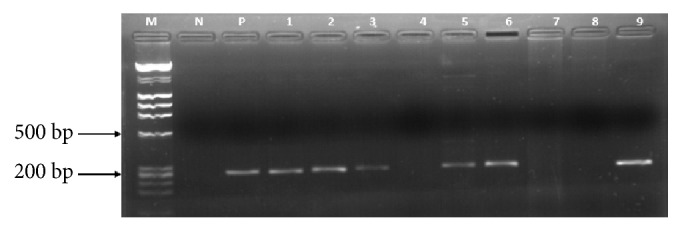
A 1% agarose gel showing the resolution of PCR amplicons generated using OIE recommended diagnostic primers for African swine fever virus. Lane M: *λ* DNA-Hind III and *ϕ*x174 DNA-Hae III mix (Finnzymes) molecular weight DNA marker, Lane N is a negative control, and Lane P is a positive control. Lanes 1, 2, 3, 5, 6, and 9 are selected positive samples with approximately 257 bp band size. Lanes 4, 7, and 8 are negative samples.

Analysis by diagnostic PCR revealed a band size of 257bp as shown in Figure 1. Ten pigs tested positive for ASF viral DNA. The positive control DNA used was from Isolate Ug12.Kampala3, previously characterized as genotype IX in our previous study [14].

### 3.2. The p72 Phylogenetic Analysis

**Figure 2:**
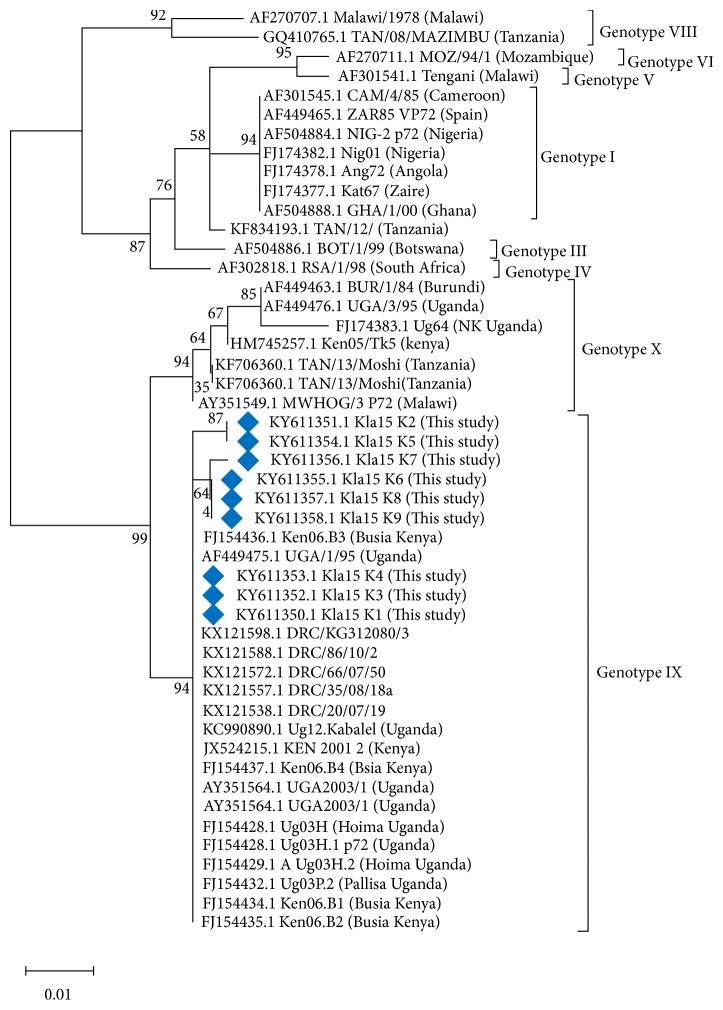
Phylogenetic tree constructed using 9 partial p72 sequences from this study and 39 from the representative genotypes from the GenBank. The evolutionary history was inferred by the maximum likelihood method based on the Kimura 2-parameter model [22]. The p72 sequences characterized from this study are denoted by Blue diamond.

A band size of 478 bp was amplified from all the samples using genotyping primers targeting the* p72* gene. Phylogenetic analysis of the partial C-terminus region of the B646L (p72) gene sequences confirmed circulation of genotype IX as shown in Figure 2

### 3.3. The p54 Phylogeny

**Figure 3:**
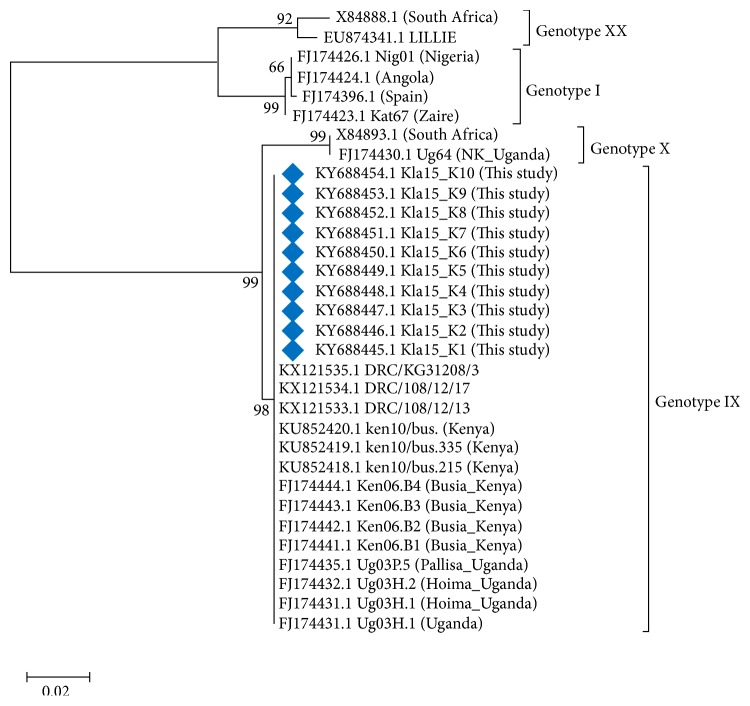
Phylogenetic tree constructed using full-length p54 sequences from this study and other sequences from the gene bank. All genotypes are represented. A maximum likelihood p54 gene tree was inferred using the K2P model of sequence evolution [22]. The analysis involved a total 32 nucleotide sequences. There were a total of 576 positions in the final dataset. Sequences from this study are denoted by Blue diamond.

A p54 genotyping PCR results showed an amplicon size of about 675bp in all ten different samples (gel not shown). Analysis of full-length p54 nucleotide sequences from this study (shown in Figure 3) confirmed that the virus responsible for 2015 outbreak belongs to genotype IX.

### 3.4. Intragenotypic Resolution (CVR) of Homogenous p72 Genotype IX

**Figure 4:**
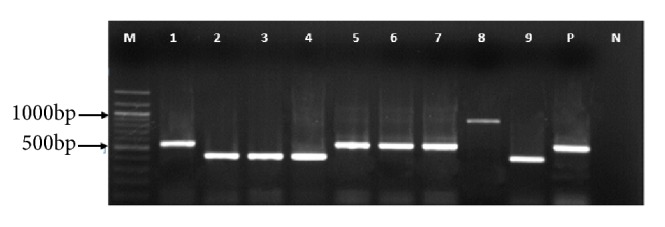
A 1% agarose gel showing the representative results obtained from amplification of the central variable region of the p72 gene of African swine fever virus using the CVR primers. Lane M: *λ* DNA-Hind III and *ϕ*x174 DNA-Hae III mix (Finnzymes) 100bp molecular weight DNA marker; Lane P is a positive control. Lanes 1, 2, 3, 5, 6, and 9 are selected positive samples with varying band sizes.

PCR amplification gave products of varying sizes (400-700bp) as shown on a 1% gel picture in Figure 4. Multiple sequence alignment of the translated CVR sequences revealed a variable number of tandem repeats within isolates from this study and among others previously isolated in Uganda and neighboring countries as shown in Table 1.

## 4. Discussion

African swine fever still remains the major constraint to pig production in Uganda with sporadic outbreaks occurring throughout the year. ASF is endemic in Uganda [7, 25, 26]. There is a need for continuous characterization of ASFV isolates during disease outbreaks in order to further understand ASF disease outbreak patterns and mapping of viral genotypes to the geographical regions they circulate within. Continuous genotyping is important in giving insight into the virus origin during every outbreak, thus improving our epidemiological understanding of the disease [25].

There is neither a cure nor vaccine currently available for ASF on market today because of the many challenges encountered by researchers [27, 28]. This makes early detection, the slaughter of infected and in-contact animals, safe carcass disposal, good sanitation, disinfection, movement controls, quarantines, and accurate diagnostic procedures of the main control measures which have a negative impact on the affected areas [15].

In this study, molecular methods were used to diagnose and characterize ASFV from domestic pigs that died of a hemorrhagic disease outbreak between August and September 2015 in Kampala. The results obtained from the present study confirm ASF outbreak in the Kanyanya and Muyenga suburbs of Kampala city.

By both B646L (p72) and E183L (p54) nucleotide sequencing, ASFV isolates from this study clustered with those in genotype IX. Both p54 and p72 genes were highly conserved among the viruses from our study and this is consistent with findings of previous studies [29].

Phylogenetic analysis revealed that the isolates from this study were closely related to those previously characterized in Democratic Republic of Congo, Uganda, Kenya, and Tanzania studies [15, 21, 30, 31]. This suggests that transportation of live pigs or their products across porous Ugandan districts and borders to Kampala, the major pork consumption area, might have been the source of the virus that caused the outbreak. However, studies that focus on ASF outbreak investigation in all countries should be encouraged and coordinated in order to enable disease reporting and ASFV genotyping.

The hypervariable B602L gene contains twelve-base pair repeat regions that code for tetrameric repeats amino acid sequences. These repeats vary in number and sequence depending on the virus genotype or even individual virus isolates [12, 16, 32]. For intragenotypic resolution of ASFV isolates, the B602L (CVR) gene was amplified in order to identify possible differences among isolates in a given genotype. CVR amplicons from this study ranged from 400bp to 800bp. The ten samples clustered in three band size groups; these are 400bp, 500bp, and 800bp as shown in Figure 4. This clearly indicated that each of these isolates had a variable number of tetrameric repeat sequences and thus each subgroup was different from each other in terms of band size.

Analysis of CVR amino acid sequences reveals tetrameric repeats that have been assigned single letter codes corresponding to each type. Tetrameric amino acid repeats that have been previously characterized in ASFV isolates include repeat code** A** = CAST/CVST/CTST,** B**=CADT/CTDT** C**=GAST/GANT,** D**=CASM,** F**=CANT,** G**= CTNT,** J**=GTDT,** K**= CTSP,** L**=YTNT,** M**=NEDT,** N**=NVDT/NVGT/NVNT,** O**=NANI/NADI/NASI,** H**=RAST,** S**=SAST,** T**=NVNT,** U**=NIDT/NTDT,** V**=NAST/NADT/NANT,** W**=SADT/SVDT, and** X**=NTDI [11, 12, 16, 31, 33].

**Figure 5:**
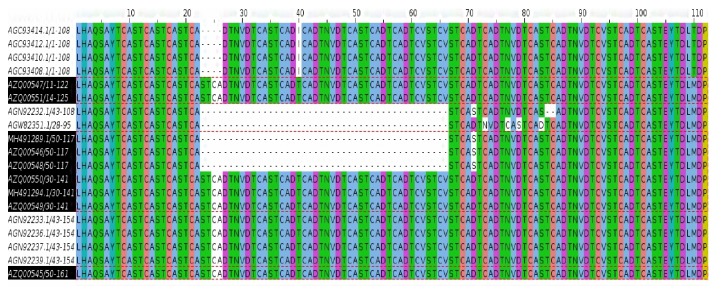
A multiple sequence alignment of the CVR amino acid sequences from this study and previous studies in Uganda and neighboring countries emphasizing tetrameric repeats of representative genotypes.

In this study, all CVR tetrameric repeats included** CAST**,** CADT**, and** NVDT** and therefore codes A, B, and N as shown in Figure 5.

Six of the viruses had 24 repeats** AAAABNABBNABBAABBNABNABA** consistent with the CVR previously characterized in Uganda [14]. However, three of our sequences indicated major deletions as they only contained 13** AAAAABNABNABA** repeats as shown in Table 1. These deletions are similar to some isolates previously characterized from Kampala Uganda [14].

B646L (p72), E183L (p54), and B602L (CVR) nucleotide sequences from this study indicate genotype IX and are probably as a result of the viruses maintained only in the domestic cycle.

The ASF viruses from this study compared to those from previous studies in Uganda show that there is a constant reintroduction of ASFV in Kampala from areas around Ugandan international borders. This is indicated by the high CVR region sequence similarity of most viruses from this study to those of previous studies done in Uganda [14]. The markedly different isolates could be as a result of a genetic mutation in the genomes of viruses already circulating in Uganda since ASF is endemic in Uganda or as a consequence of animal movements. This could probably be true because of the continuous uncontrolled pig movement from all over Uganda to Kampala where pork is mostly consumed.

The similarity of the ASFV isolates from this study with those recently documented in the previous studies indicates that there is a continuous movement of pigs and pig products from most local places in Uganda to Kampala for various reasons most especially trade. This explains the sporadic outbreak of ASF with the same ASFV genotypes circulating in Kampala, Uganda, and other close neighboring countries like Kenya, Tanzania, and Democratic Republic of Congo [21, 30, 31]. This, therefore, calls for regulation or close monitoring of movement of domestic pigs into Kampala and in addition setting up proper biosecurity measures at the city abattoirs in Kampala to prevent the spread of ASF from the abattoirs into farms in the city suburbs. Outbreaks in the suburban areas of Kampala are of a significant economic impact because many of these peri-urban farms have larger numbers of pigs as opposed to rural farmers with free-ranging pigs.

## 5. Conclusions

This study confirmed that ASFV genotype IX was responsible for the reported outbreak in Kampala. Some of the viruses from this study are closely related to those previously isolated in Uganda; however, some are rather different, due to changes in the CVR gene region. Phylogenetic analysis shows that sequences from this study clustered closely with those from Busia (Kenya) and DRC. This probably indicates uncontrolled movement of pigs, by pig traders from the rural to urban areas of Uganda.

## Figures and Tables

**Table 1:** The amino acid sequence of the tetrameric repeats that constitute the central variable region (CVR) of the B602L gene characterized in this study. Key: A (CAST); B (CADT), and N (NVDT).

Accession numbers	*Isolate name*	Countr*y*	CVR variant	CVR amino acid	No of repeats	P72 Genotype	Reference
**AZQ00545**	**Kla15.Kampala1**	**Uganda**	**1**	**AAAABNABBNABBAABBNABNABA**	**24**	**IX**	**This study**
**MH491289**	**Kla15.Kampala2**	**Uganda**	**2**	**AAAAABNABNABA**	**13**	**IX**	**This study**
***AZQ00549***	**Kla15.Kampala4**	**Uganda**	**1**	**AAAABNABBNABBAABBNABNABA**	**24**	**IX**	**This study**
***AZQ00546***	**Kla15.Kampala6**	**Uganda**	**2**	**AAAAABNABNABA**	**13**	**IX**	**This study**
**MH491294**	**Kla15.Kampala5**	**Uganda**	**1**	**AAAABNABBNABBAABBNABNABA**	**24**	**IX**	**This study**
**AZQ00548**	**Kla15.Kampala10**	**Uganda**	**1**	**AAAAABNABNABA**	**13**	**IX**	**This study**
**AZQ00550**	**Kla15.Kampala7**	**Uganda**	**1**	**AAAABNABBNABBAABBNABNABA**	**24**	**IX**	**This study**
**AZQ00551**	**Kla15.Kampala8**	**Uganda**	**1**	**AAAABNABBNABBAABBNABNABA**	**24**	**IX**	**This study**
**AZQ00547**	**Kla15.Kampala9**	**Uganda**	**1**	**AAAABNABBNABBAABBNABNABA**	**24**	**IX**	**This study**
*AGN92237*	Ug11.Mpigi	Uganda	1	AAAABNABBNABBAABBNABNABA	24	IX	[14]
*AGN92239*	Ug10.Moyo1	Uganda	1	AAAABNABBNABBAABBNABNABA	24	IX	[14]
*AGN92233*	Ug12.Kabale1	Uganda	1	AAAABNABBNABBAABBNABNABA	24	IX	[14]
*AGN92236*	Ug10.Adjumani2	Uganda	1	AAAABNABBNABBAABBNABNABA	24	IX	[14]
*AGW82351*	Uga12.Nakasongola	Uganda	2	AAAAABNABBNABA	13	IX	[14]
*AGN92232*	Ug13.Kampala1	Uganda	2	AAAAABNABBNABA	13	IX	[14]
AGC93414	Ken11/KakSP	Kenya	1	AAAABNABBNABBAABBNABNABA	24	IX	[18]
AGC93412	Ken11/Kia2.1	Kenya	1	AAAABNABBNABBAABBNABNABA	24	IX	[18]
AGC93410	Ken10/Kis028	Kenya	1	AAAABNABBNABBAABBNABNABA	24	IX	[18]
AGC93408	Ken10/KAKFA1	Kenya	1	AAAABNABBNABBAABBNABNABA	24	IX	[18]

## Data Availability

The ASF virus nucleotide and amino acid accession numbers obtained and used to support the findings of this study have been deposited in the GenBank [https://www.ncbi.nlm.nih.gov/nuccore/?term=Molecular+characterization+of+African+swine+fever+viruses+from+the+++++++++++++August-September%2C+2015+outbreak+in+Kampala%2C+Uganda].

## Additional Points

*Recommendations. *ASF continues to remain endemic in Uganda; therefore continuous genetic characterization should be encouraged in order to contribute more sequence data that can be used in understanding the molecular epidemiology of ASF and in designing of appropriate control measures. ASF outbreaks in Uganda are constantly happening, and a thorough outbreak investigation would probably bring about a proper understanding of the ASF epidemiology in Uganda.
